# Research Progress in the Mechanisms of Resistance to Biotic Stress in Sweet Potato

**DOI:** 10.3390/genes14112106

**Published:** 2023-11-20

**Authors:** Yinghui Yang, Yanqi Chen, Yuxin Bo, Qingchang Liu, Hong Zhai

**Affiliations:** Key Laboratory of Sweetpotato Biology and Biotechnology, Ministry of Agriculture and Rural Affairs/Beijing Key Laboratory of Crop Genetic Improvement/Laboratory of Crop Heterosis and Utilization, Ministry of Education, College of Agronomy & Biotechnology, China Agricultural University, Beijing 100193, China; yyh1105038236@163.com (Y.Y.); cyq970131@163.com (Y.C.); s20213010032@cau.edu.cn (Y.B.); liuqc@cau.edu.cn (Q.L.)

**Keywords:** sweet potato, fungi, nematodes, insects, virus, bacteria, resistance mechanisms

## Abstract

Sweet potato (*Ipomoea batatas* (L.) Lam.) is one of the most important food, feed, industrial raw materials, and new energy crops, and is widely cultivated around the world. China is the largest sweet potato producer in the world, and the sweet potato industry plays an important role in China’s agriculture. During the growth of sweet potato, it is often affected by biotic stresses, such as fungi, nematodes, insects, viruses, and bacteria. These stressors are widespread worldwide and have severely restricted the production of sweet potato. In recent years, with the rapid development and maturity of biotechnology, an increasing number of stress-related genes have been introduced into sweet potato, which improves its quality and resistance of sweet potato. This paper summarizes the discovery of biological stress-related genes in sweet potato and the related mechanisms of stress resistance from the perspectives of genomics analysis, transcriptomics analysis, genetic engineering, and physiological and biochemical indicators. The mechanisms of stress resistance provide a reference for analyzing the molecular breeding of disease resistance mechanisms and biotic stress resistance in sweet potato.

## 1. Introduction

Sweet potato (*I. batatas* (L.) Lam.) is a natural heterozygous hexaploid crop that belongs to the Convolvulaceae family within the *Ipomoea* genus and *Batatas* species. Sweet potato is grown in over 120 countries and regions, with an annual planting area of approximately 8.063 million hectares and an annual total yield of approximately 91.95 million tons, ranking eighth in the global grain production (FAO, 2020) [1]. China is the largest producer of sweet potato, with an annual planting area of approximately 2.379 million hectares, accounting for 29.51% of the global total, and an annual total yield of about 53.25 million tons, accounting for 57.91% of the world’s production (FAO, 2020). Sweet potato is used as an important food, feed, industrial raw material, and new energy crop because of its rich contents of starch, soluble sugar, dietary fiber, and various trace elements. In nature, sweet potato growth is not only inhibited by abiotic stresses (such as salt and drought) but also by biotic stresses such as bacteria, fungi, viruses, and stem nematodes, which seriously affect the yield and quality of the crop. The selection and utilization of disease-resistant varieties are the most efficient ways to reduce losses caused by biotic stress [2,3]. At present, there are limited disease-resistant germplasm resources available for sweet potato. Therefore, it is essential for sweet potato disease-resistance molecular breeding to explore disease-resistance genes and analyze their molecular regulatory mechanisms.

## 2. Progress on the Molecular Mechanism of Biological Stress Resistance in Plants

Plants rely on their innate immunity to sense and defend themselves against potential pathogens. The plant innate immune system is comprised of two interconnected systems: pathogen-associated molecular patterns, PAMP-triggered immunity (PTI), and effector-triggered immunity (ETI). PAMP-triggered immunity constitutes the first line of defense against pathogen-associated molecular patterns in the plant immune system [4]. When plants are infected by pathogens, most invasive pathogens are recognized by a transmembrane protein called pattern recognition receptor (PRRS). After recognizing the pathogen, PRRs convey immune signals downstream through the cytosolic receptor kinases Botrytis-induced kinase1 (BIK1) [5]. In addition, the Mitogen-activated protein kinase (MAPK) cascade and Calcium-dependent protein kinase (CDPK) can activate the corresponding immune responses, such as the buildup of reactive oxygen species (ROS) and callose accumulation [6]. PTI can also be activated by endogenous plant signals and cell wall-associated kinases (WAKs) that are released upon pathogen invasion [7,8]. As pathogens continue to evolve, some pathogen effectors can avoid detection by PTI in plants [9,10,11]. In response, a second layer of immune defense, ETI, has evolved in plants to protect against these pathogens [12,13]. The R genes are resistance genes in plants, and they mostly encode a type of protein containing a leucine-rich repeat (NLR) domain. These R proteins can directly or indirectly recognize pathogen-sensing toxic effector proteins in cells, triggering immune responses such as stomatal closure, ROS accumulation, and programmed cell death initiation, thereby rendering plants resistant to diseases [14,15,16,17].

## 3. Progress on the Molecular Mechanism of Biological Stress Resistance in Sweet Potato

The growth of sweet potato is affected by bacteria, fungi, viruses, nematodes, and insects [2,3]. Bacterial infections usually infiltrate through wounds or natural pores in various parts of sweet potato, damaging the cell structure and causing metabolic abnormalities [18,19,20,21,22,23,24,25]. This can directly endanger the development of sweet potato and seriously affect their yield and quality [26]. Sweet potato is an autopolyploid crop with a highly complex genetic background, large number of chromosomes, and self-incompatibility, which directly leads to the difficulties in sweet potato breeding. At present, there are few reports on the genome sequencing of sweet potato cultivars [27,28] and its related wild species, *Ipomoea trifida* [29] and *I. triloba* [30]. Due to the imperfect genome information and complex genetic background of sweet potato, the development of sweet potato biotechnology lags behind other plants [31]. At present, there are some reports on the cloning, functional analysis, and molecular mechanisms of disease resistance-related genes in sweet potato.

### 3.1. Exploration of Resistance Genes in Sweet Potato for Fungal Disease and Their Mechanisms

**Figure 1:**
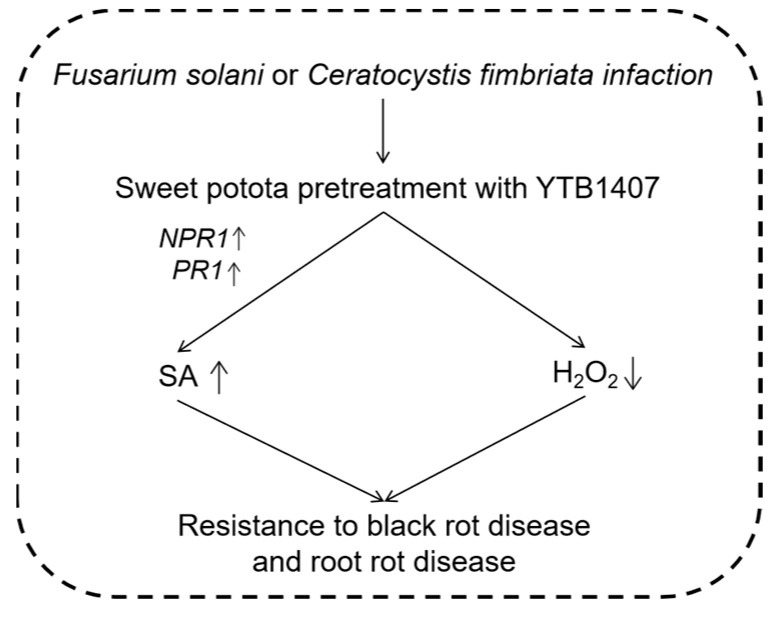
The mechanism of resistance to root rot and black rot diseases in sweet potato.

**Figure 2:**
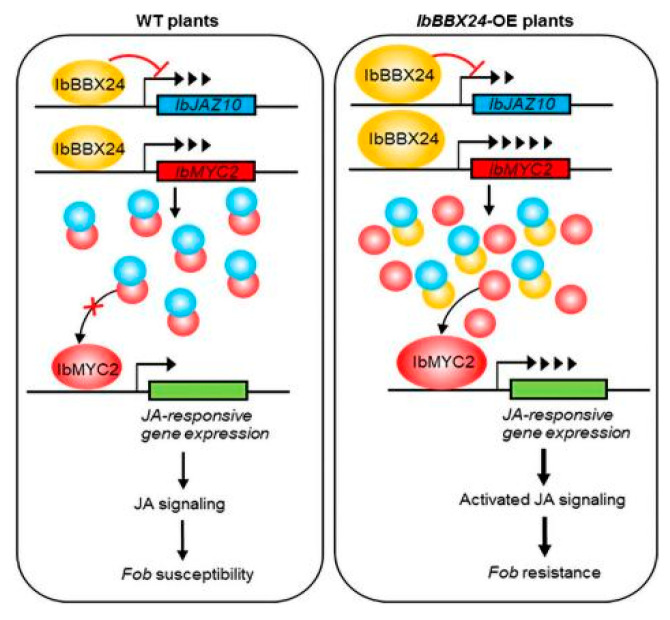
The mechanism of resistance to Fusarium wilt in sweet potato [38].

During plant growth and post-harvest storage, the pathogenic fungus *Ceratocystis fimbriata*, which causes the black rot in sweet potato, severely damages crops. Sweet potato infected with *C. fimbriata* contain ipomeamarone, which can cause poisoning of livestock. Using sick sweet potato as raw material delays the fermentation process and reduces the yield and quality of alcohol, thereby seriously restricting the development of the sweet potato industry. The thionin peptide from this plant demonstrated antifungal activity against *C. fimbriata*. An α-hordothionin (*α-HT*) gene from barley, which was placed downstream of a strong constitutive promoter of E12Ω or the promoter of a sweet potato gene for β-amylase of storage roots, was introduced into sweet potato cv. Kokei No. 14. The gene exhibited high levels of expression in both leaves and storage roots. Compared to the control, the transgenic sweet potato exhibited reduced yellowing upon infection, and the storage roots showed reduced lesion areas around the site inoculated with *C. fimbriata* spores [32]. The endophytic *Bacillus amyloliquefaciens* YTB1407 has previously been reported to promote the growth of sweet potato. Researchers used YTB1407 suspension pretreatment of sweet potato in both in vitro and pot trials. The results indicated that, compared to the control, pretreatment with YTB1407 enhanced resistance against root rot disease (*Fusarium solani* Mart. Sacc.f. sp. *batatas* McClure) and black rot disease (*C. fimbriata* Ell. &Halst). The pretreatment activated the expression of the salicylic acid (SA)-responsive *PR-1* gene, increased the SA content, and reduced hydrogen peroxide (H_2_O_2_) in the host to resist *F. solani* infection. Additionally, it enhanced the expression levels of SA-responsive *NPR1* and *PR1* genes and increased the SA content to resist *C. fimbriata* infection [33] (Figure 1). Fusarium wilt is a disease caused by *Fusarium oxysporum* f. sp. *batatas* (*Fob*). The leaves of diseased sweet potato plants leaves turn yellow and fall off, the stem vascular bundles become brown, and eventually, the whole plant dies, resulting in yield losses of 10% to 50% in sweet potato [34,35]. Lin et al. used sweet potato varieties JinShan57 (high resistance) and XinZhongHua (high sensitivity) to identify gene families related to Fusarium wilt by RNA-seq sequencing. They found that the SA and JA pathways, which showed more differential gene enrichment, were also involved in the regulation of disease-resistance mechanisms. The R gene is involved in this process and triggers the ETI pattern to defend against pathogens in this fungal disease [35]. Jing et al. selected the significant differential gene *IbMAPKK9* from the sweet potato transcriptome data. It is expressed in the roots, stems, leaves, and petioles of sweet potato in response to *F. oxysporum* infection. The transient expression of *IbMAPKK9* caused the upregulation of five genes (*NtPAL4*, *NtICS1*, *NtNPR1*, *NtNPR3* and *NtNPR5*) related to the SA synthesis and signal transduction pathways within 48 h. It is speculated that *IbMAPKK9* affects plant resistance by mediating the SA signaling pathway [36]. Li et al. cloned the *IbSWEET10* gene from the sweet potato line ND98. Overexpression of *IbSWEET10* significantly enhanced sweet potato’s resistance to Fusarium wilt, exhibiting better growth and a significant reduction in sugar content after infection compared with the control. Conversely, the RNAi plants showed the opposite results. Therefore, the reduction in sugar content caused by *IbSWEET10* overexpression is the major reason for the enhanced *F. oxysporum* resistance of transgenic plants [37]. When the B-box (BBX) family transcription factor gene *IbBBX24* was overexpressed in sweet potato, jasmonic acid (JA) accumulation increased, whereas silencing of this gene decreased JA levels. Overexpression of *IbBBX24* significantly increased Fusarium wilt disease resistance, suggesting that JA response plays a crucial role in regulating Fusarium wilt resistance in sweet potato. IbBBX24 regulates JA responses by antagonizing the JA signaling repressor *IbJAZ10*, which relieves IbJAZ10′s repression of *IbMYC2*, a JA signaling activator. Overexpression of *IbBBX24* also led to an increase in the yield of sweet potato. These findings indicate that IbBBX24 plays a pivotal role in regulating JA biosynthesis and signaling pathway, as well as in increasing Fusarium wilt resistance and yield in sweet potato [38] (Figure 2). Liu et al. used the *F. oxysporum f.* sp. *batatas* to infect sweet potato cultivars Eshu11 (high resistance to Fusarium wilt) and Lizixiang (high sensitivity to Fusarium wilt). The expression of the *IbWRKY7* gene was significantly upregulated in the two cultivars. In Eshu11, the expression of the gene reached its maximum at 4 h after infection, whereas in Lizixiang, it reached its maximum at 96 h post-infection. In the early stages of infection, the expression of *IbWRKY7* in the resistant cv. Eshu11 was higher, indicating that *IbWRKY7* in Eshu11 responded more quickly to the pathogen infection. *IbWRKY7* may play an important role in the early response to pathogen infection in sweet potato [39]. Liu et al. cloned the *IbERF1* gene from Eshu11 and used real-time quantitative PCR to analyze it. The results showed that the expression of *IbERF1* in Eshu11 significantly increased after 2 and 4 h of pathogen infection. Treating with methyl jasmonate (MeJA) and ethylene (Eth) for 2, 4, and 12 h significantly increased the expression of the gene. Therefore, *IbERF1* might regulate the resistance to Fusarium wilt in sweet potato by mediating the MeJA/Eth signaling pathway [40].

The use of endophytic bacteria for controlling sweet potato diseases is one of the important ways of biological control. Li et al. isolated a strain of *Bacillus subtilis* from the soil that displayed antagonistic effects against Fusarium wilt in sweet potato. The detection of physiological and biochemical indicators revealed that the Bacillus subtilis HAAS01 strain could enhance the production of endogenous hormones in sweet potato and was associated with the upregulation of defense enzymes and related gene expression. This collective action helps to combat the infection of plant diseases [41]. Furthermore, Hossain et al. developed a simple method for the green synthesis of AgNPs from Bacterium *Pseudomonas rhodesiae* culture supernatant, which showed potent antibacterial activity against the pathogen *D. dadantii*. Therefore, this method can be used to produce healthy sweet potato crops [42].

Root rot caused by *Fusarium solani*, manifests in sweet potato as black and rotten root parts, resulting in small size or the inability to form potato pieces, ultimately affecting the yield and quality of sweet potato roots. Root rot is a major post-harvest disease limiting sweet potato production. Pan et al. found that a cinnamaldehyde (CA) concentration of 0.075 g/L significantly inhibited the viability of *F. solani* conidia. CA vapor at a concentration of 0.3 g/L in the air effectively managed the growth of *F. solani* in sweet potato during a 10-day storage period at 28 °C, safeguarding the soluble sugar and starch in the storage root from being depleted by the fungus [43].

### 3.2. Exploration of Resistance Genes in Sweet Potato for Nematodes and Their Mechanisms

Sweet potato stem nematode disease is caused by the *Ditylenchus destructor*, which is a plant parasitic nematode [44]. Stem nematodes invade the masses from the stems, causing them to rot from the inside, and the tissue to lose water, resulting in a chequered pattern of black and white in both stems and internal tissue. Stem nematodes pose a significant threat to sweet potato production by seriously affecting yield and quality, typically resulting in a 20 to 50% reduction in yield or even complete crop failure.

**Figure 3:**
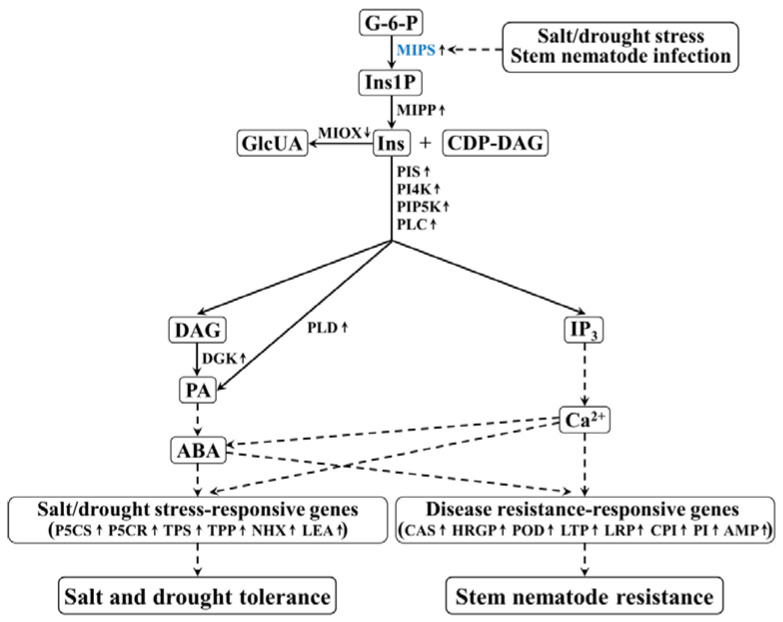
The mechanisms of resistance to stem nematode disease in sweet potato [51].

Si et al. conducted genome screening analysis, combining transcriptome analysis of sweet potato cultivars or lines Tengfei (sweet potato stem nematodes susceptible cultivar), JK20 (sweet potato stem nematodes resistant line) infected with stem nematodes, and Santiandao (*C. fimbriata* susceptible cultivar), JK142 (*C. fimbriata* resistant line) infected with *C. fimbriata*, respectively. A total of 11 differentially expressed NBS genes were found in Tengfei and JK20 after infection by stem nematodes, and 19 differentially expressed NBS genes were found in Santiandao and JK274 after infection by *C. fimbriata*, respectively. These results will provide a reference for the exploration of sweet potato disease resistance genes [45]. Yan et al. used sweet potato cv. American Red (♂) resistant to stem nematode, and cv. Xuzishu8 (♀) susceptible to stem nematode to generate a population of 274 F_1_ progenies. In an inoculation experiment with stem nematodes, the extended resistance heritability of sweet potato was 75.7%, which was mainly controlled by genetic factors. The 10 QTLs obtained were tightly linked to stem nematode resistance in sweet potato and selected out the key candidate gene *itf13g19570* [46]. Cai et al. introduced the sweet potato storage protein gene *SpTI-1* into sugar beets and found that the growth and development of female nematodes in eight rooting clones were inhibited when infected with sugar beet cyst nematodes. This indicates that the growth and development of nematodes are related to trypsin inhibitor activity [47]. Fan et al. constructed an RNAi interference vector based on the unc-15 sequence of sweet potato stem nematodes and introduced it into the sweet potato variety Xushu22 using *Agrobacterium-mediated* methods. The resistance of transgenic sweet potato to stem nematode diseases has improved [48]. Oryzacystatin-I (OCI) protein is a proteinase inhibitor, that inhibits the proteinase activity in the nematode intestinal canal and prevents the assimilation of proteins. Gao et al. transferred the *OCI* gene into sweet potato cultivars Xushu 18 and Lizixiang, and overexpression of *OCI* significantly enhanced the resistance of the transgenic sweet potato plants to stem nematode disease [49,50]. Zhai et al. found that under field conditions, sweet potato overexpressing the *IbMIPS1* gene significantly enhanced the stem nematode resistance, salt tolerance, and drought resistance of transgenic sweet potato. Transcriptome and real-time quantitative PCR analysis showed that under salt, drought, and stem nematode stresses, the overexpression of *IbMIPS1* upregulated genes involved in inositol biosynthesis, phosphatidylinositol (PI) and abscisic acid (ABA) signaling pathways, stress response, photosynthesis, and ROS clearance systems. The corresponding content of resistance-related substances significantly increased, whereas the content of substances such as malondialdehyde (MDA) and H_2_O_2_ significantly decreased [51] (Figure 3).

The southern root-knot nematode (SRKN), *Meloidogyne incognita* is a typical parasitic nematode that affects sweet potato, leading to a significant reduction in crop yield and commercial value. Based on the identification of resistance in F_1_ isolated populations constructed from root-knot nematode-resistant cv. J-Red and susceptible cv. Choshu, as well as genome-wide association analysis, Obata et al. discovered that resistance to SP2, which is the major race in areas with high sweet potato production in Japan, could be regulated by two loci in sweet potato. Subsequently, selective DNA markers were developed using the SNPs identified on Chr03 and Chr07, to screen for resistant plants. When combined with two selective DNA markers, the likelihood of selecting SRKN-SP2-resistant plants was approximately 70% [52].

### 3.3. Exploration of Resistance Genes in Sweet Potato for Pest and Their Mechanisms

Sweet potato pests are one of the significant factors that affect the normal growth of sweet potato, severely impacting its yield and quality. Sweet potato pests can be categorized as either aboveground or underground pests. Aboveground pests include wheat moths, purslanes, tobacco whiteflies, red spiders, sweet potato corn borers, and sweet potato weevil [53,54,55,56,57,58,59]. The larvae of these pests feed on the young sweet potato leaves. In severe cases, they can consume all the leaves, leaving only the old stems, which leads to poor plant growth and impedes the development of sweet potato storge roots, thereby reducing the yield of sweet potato. Underground pests include larvae, sweet potato ant weevils, golden needles, small ground tigers, and sweet potato leaf beetles [60,61,62]. These pests primarily target the roots of sweet potato, resulting in a shortage of sweet potato plants and increasing the decay rate of sweet potato during the field and storage periods, severely affecting the yield and quality of sweet potato. Currently, the prevention and control of sweet potato pests primarily focuses on prevention, employing comprehensive physical, chemical, and biological control methods.

**Figure 4:**
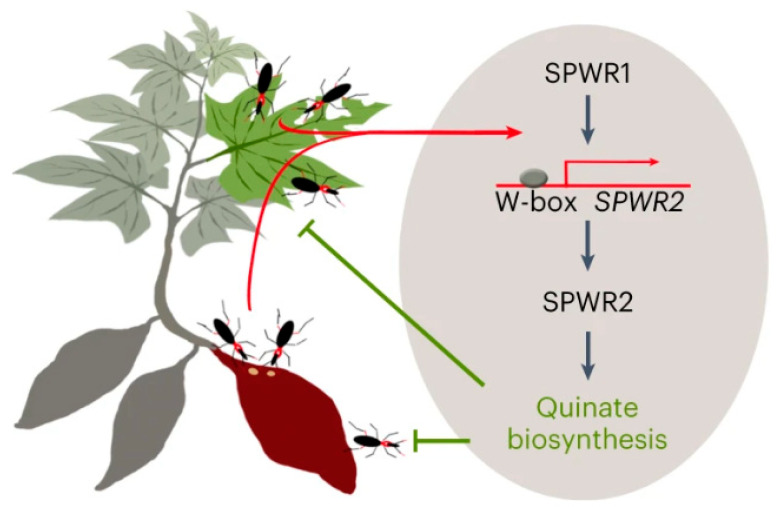
The mechanisms of resistance to sweet potato weevils in sweet potato [63].

Sweet potato weevils (SPWs) *Cylas formicarius* (Fabricius) are one of the most significant challenges in sweet potato production in the tropical and subtropical regions. The adults survive by feeding on the leaves and storge roots of sweet potato, whereas the larvae mainly damage the stem base and storge roots, which results in a significant reduction in the quality and edibility of sweet potato. SPWs are listed as international quarantine pests causing detrimental economic and environmental effects. Liu et al. obtained two sweet potato germplasms, N73 and N28, with high SPW resistance through the screening and identification of 282 sweet potato germplasms, including 208 germplasms from Guangdong, Guangxi, and Hainan Provinces and 74 cultivars from East Asia. The F_1_ population (*n* = 240) was obtained by crossing a major sweet potato cultivar G87 (♀) with N73 (♂). Using the F_1_ population for genetic map construction, two important resistance genes, *SPWR1* and *SPWR2*, were obtained through map cloning and functional identification methods. They play crucial roles as regulatory factors in natural defense against SPWs. Further analysis revealed that the SPW-induced WRKY transcriptional factor SPWR1 could directly activate the expression of *SPWR2*. These findings provide new insights into the molecular mechanism underlying sweet potato-SPW interactions. [63] (Figure 4). Zhang et al. discovered that temperature is an important factor affecting the growth activity of sweet potato weevils. The growth and elimination dynamics of sweet potato showed a significant negative correlation with the average temperature but no obvious correlation with precipitation and relative humidity. The difference in sweet potato varieties and the depth of sweet potato will significantly affect the growth and elimination of sweet potato weevils and will directly cause different degrees of harm to sweet potato weevils [64].

### 3.4. Exploration of Resistance Genes to Sweet Potato Virus Diseases and Their Mechanisms

Sweet potato virus is the most devastating viral disease in sweet potato, and it was found that most sweet potato viruses appear as two or more complex forms of the virus, and the form of infection alone is less common. Affected by changes in the ecological environment, the increasingly serious virus disease causing substantial yield losses worldwide [65]. At present, sweet potato production is mainly constrained by sweet potato virus disease (SPVD) caused by the co-infection with sweet potato chlorotic stunt virus (SPCSV) and sweet potato feathery mottle virus (SPFMV). However, the current understanding of sweet potato responses to SPCSV and SPFMV at the molecular level remains very limited.

Bednarek et al. analyzed mRNA and small RNA (sRNA) data from the susceptible variety ‘Beaured’ infected with SPVD and found that pathways related to stress response and signal transduction were significantly affected by the virus infection. In the late stage of co-infection between SPCSV and SPFMV, the sRNA components of these pathways were mainly affected. Further analysis revealed that several new microRNAs were responsive to viral infection, some of which were predicted to target resistance genes rich in leucine repeat sequences (NBS-LRR) at nucleotide binding sites. They also found that the downregulation of the defense response pathway mediated by SA could partially explain the susceptibility of the ‘Beaured’ variety to SPVD [66]. Zhang et al. examined the transcriptomes of SPVD-infected and uninfected sweet potato cv. Wanshu No.8 and discovered that some genes associated with photosynthesis, starch and sucrose metabolism, flavonoid biosynthesis, and carotenoid biosynthesis were downregulated following SPVD infection, while other genes involved in monolignol biosynthesis, zeatin biosynthesis, trehalose metabolism, and linoleic acid metabolism were upregulated. Remarkably, after SPVD infection, the expression of key genes associated with pathogenesis and plant defense was significantly induced or suppressed. These findings provide some insights into the pathogenesis and defense mechanisms of sweet potato against SPVD [67]. The CRISPR-Cas13 technique was used to target one of its important pathogenesis-related factors (i.e., SPCSV-RNase3) to enhance SPVD resistance. Yu et al. found that RfxCas13d, driven by the pCmYLCV promoter for the expression of gRNAs, demonstrated higher RNA-targeting activity than that driven by the pAtU6 promoter. Additionally, the targeting of SPCSV-RNase3 using the LwaCas13a system inhibited its RNA-silencing suppressor activity and restored its RNA-silencing activity in *N. benthamiana* leaf cells. Compared to the wild type, transgenic *N. benthamiana* plants carrying an RNase3-targeted LwaCas13a system displayed enhanced resistance against turnip mosaic virus TuMV-GFP and cucumber mosaic virus CMV-RNase3 co-infection, while transgenic sweet potato plants carrying an RNase3-targeted RfxCas13d system exhibited substantially improved SPVD resistance [68].

### 3.5. Exploration of Resistance in Sweet Potato for Bacteria Disease and Their Mechanisms

The infection process of bacterial wilting in sweet potato generally begins with wounds in the rhizome or the natural orifice of sweet potato. When bacterial wilt pathogen infects sweet potato, it immediately colonizes the cortex and intercellular space, then reproduces extensively in plant ducts and nearby tissues, secretes extracellular polysaccharides to block ducts, destroys surrounding tissues, and finally leads to plant wilt and death. Sweet potato stem rot can occur in all periods, from sweet potato sowing to harvest, mainly affecting the roots, stems, and petioles of sweet potato, and the leaves are also infected in severe cases [69,70,71]. Yuan et al. employed RAPD technology to analyze the F_1_ (Jinshan 57 × Jinshan 630) population’s resistant and susceptible pools, and screened and obtained a primer (S213-500) from among 320 primers. The PCR amplified product of this primer was found to be as-sociated with the bacterial wilt resistance gene [72]. Wang et al., found that phenylalamine ammonia lyase (PAL) and peroxidase (POD) activities increased and the content of lignin and chlorogenic acid also increased in sweet potato cv. Xiangshu75-55 (*Pseudomonas solanacearum* resistant cultivar) compared with Shengli100 (*P. solanacearum* susceptible cultivar) after *P. solanacearum* infection [73]. Yu et al. treated the sweet potato leaves from Xiangshu75-55 and Shengli100 with different concentrations of SA for 3 d and found that SA improved the activity of superoxide dismutase (SOD) and catalase (CAT), reduced the activity of POD and MDA content and reduced the accumulation of H_2_O_2_ in leaves under *Ralstonia solanacearum* infection. This results showed that 5 mmol · L^−1^ SA was the best optimum concentration to improve the disease resistance of sweet potato [74].

## 4. Expectation

As an emerging cash crop, sweet potato is becoming increasingly valuable due to its multiple uses and anticancer health benefits, and the economic benefits of its cultivation are significant. However, the increasing numbers of sweet potato fungi, nematodes, insects, viruses, and bacteria are becoming increasingly serious, limiting the production and variety improvement of sweet potato. The development of bioinformatics and sequencing technologies has greatly facilitated the study of biotic stress resistance in sweet potato, and less genomic information has been obtained from the sweet potato variety Tai.6 and two closely related wild species [27,28,29,30]. In addition, some sweet potato biotic stress resistance genes have been screened, and some gene functions have been validated by sequencing analysis of the genome and transcriptome.

At present, the research on the biotic stress in sweet potato are still at the stage of continuous exploration and research. With further improvements in sweet potato genome information and the development of modern biotechnology, the mining of important trait genes in sweet potato will be accelerated. The combination of molecular technology breeding with conventional breeding technology will promote the development of new high-yield, high-quality, stress-resistant sweet potato germplasms and variety selection, which will greatly promote the development of sweet potato breeding.

## Data Availability

Not applicable.
